# Praxiseinsatz Elektronischer Patientenakten: Erkenntnisse aus 2 Versorgungsprojekten in Zentren für Seltene Erkrankungen

**DOI:** 10.1007/s00103-022-03599-8

**Published:** 2022-10-24

**Authors:** Asarnusch Rashid, Daniela Choukair, Christoph Bauer, Melanie Ullrich, Tim Maisch

**Affiliations:** 1 Zentrum für Telemedizin Bad Kissingen, Bad Kissingen, Deutschland; 2 Zentrum für Seltene Erkrankungen, Universitätsmedizin Heidelberg, Heidelberg, Deutschland; 3 Zentrum für Seltene Erkrankungen – Referenzzentrum Nordbayern, Universitätsklinikum Würzburg, Würzburg, Deutschland; 4 PIB & CAP, Abteilung Dermatologie, Universitätsklinikum Regensburg, Franz-Josef-Strauss-Allee 11, 93053 Regensburg, Deutschland

**Keywords:** eHealth, Vernetzung, Telemedizin, Telekonsile, Digitalisierung, eHealth, Networks, Telemedicine, Teleconsultation, Digitization

## Abstract

Elektronische Patientenakten (EPA) bieten zahlreiche Chancen für die digitale Vernetzung der Leistungserbringer untereinander und für die digitale Kommunikation mit den Patienten. Für Menschen mit Seltenen Erkrankungen (SE) können sich dadurch verschiedene Vorteile ergeben, wie eine frühere Diagnose und eine gezieltere Behandlung z. B. auf Grundlage eines multiprofessionellen Fallmanagements. Für die Patientenversorgung und Forschung bei Seltenen Erkrankungen kann eine EPA die Daten der Patienten strukturiert erfassen und darauf aufbauend die Arbeitsabläufe von der Anmeldung über die Aufnahme bis zu Behandlung und Monitoring digital abbilden. Für das Gesundheitswesen erhofft man sich durch EPA eine Kostenersparnis, da Diagnose- und Behandlungsprozesse zielgerichteter angeboten und unnötige Untersuchungen und Termine reduziert werden können.

In 2 Pilotprojekten konnten erste Erfahrungen mit EPA für Menschen mit Seltenen Erkrankungen gesammelt werden. Die Projekte „BASE-Netz“ und „TRANSLATE-NAMSE“ haben in Zusammenarbeit mit mehreren Leistungserbringern die Anforderungen an eine EPA erfasst, die technische und rechtliche Machbarkeit aufgezeigt und die Praktikabilität für Leistungserbringer und Patienten untersucht. Während die Patienten überwiegend positive Resonanz zeigten, erwies sich die Anbindung der niedergelassenen Arztpraxen als Herausforderung. Vereinfachend könnte hierbei zukünftig der Ausbau der Telematikinfrastruktur wirken. Unerlässlich sind stetige Aufklärungen und Transparenz, um insbesondere über datenschutzrechtliche Fragen zu informieren. Auch sollten Schulungen und Unterstützung angeboten werden, um die digitalen Kompetenzen der Patienten zu fördern.

## Abstract (en)

An electronic patient record offers opportunities for digital networks between medical care providers and for the digital communication between health service providers and their patients. Patients with rare diseases benefit from a diagnosis and treatment information at an early stage and receive precise treatment on the basis of multiprofessional case management. Regarding the patient care and medical research in rare diseases, electronic patient records can help to collect all data in a structured manner and to digitally map the workflows in registration, admission, diagnosis, and treatment. This can reduce costs in our healthcare system, as diagnosis and treatment can be targeted better at the patients and unnecessary medical examinations can be reduced.

In two pilot projects, first experiences with electronic patient records for patients with rare diseases were gathered. In cooperation with several medical care providers, the projects BASE-Netz and TRANSLATE-NAMSE analyzed the requirements of an electronic patient record, demonstrated the technical and legal feasibility, and evaluated the practicability for medical care providers and patients. The participating centers for rare diseases see benefits in the structured registration of the patients and the simplification of cross-institutional patient management, as patients can fulfil more tasks on their own and the health professionals can easily share data. The development of the Telematikinfrastructure of the Gematik offers opportunities to ease the digital connection between doctors’ offices and the center for rare diseases. In particular, constant clarification and transparency are essential in order to provide information on data protection issues. Training and support should also be provided to promote patients‘ digital skills.

## Einleitung

In Deutschland leben Schätzungen zufolge derzeit ca. 4 Mio. Patienten mit einer Seltenen Erkrankung (SE), definiert durch eine Prävalenz kleiner als 1:2000 [1, 2]. Der Bekanntheitsgrad der einzelnen SE ist unter Ärzten gering, zumal eine Diagnosefindung häufig durch ein breites phänotypisches Spektrum mit Beeinträchtigung mehrerer Organsysteme erschwert wird. Viele Patienten mit einer SE durchlaufen daher jahrelange Odysseen von Arzt zu Arzt, bis die korrekte Diagnose gestellt wird. So geht oft wertvolle Zeit für eine wirkungsvolle Therapie verloren [3].

Die Versorgung von Menschen mit SE erfordert aufgrund der Seltenheit der Erkrankungen, ihrer individuellen Besonderheiten und des Multiorganbefalls eine gemeinsame Fallbeurteilung durch ein erfahrenes, interdisziplinäres und multiprofessionelles Team. Das Nationale Aktionsbündnis für Menschen mit SE (NAMSE) hat 2013 einen Aktionsplan veröffentlicht und damit entsprechend ausgestattete, sowohl ambulant als auch stationär arbeitende Fachzentren (Typ-B-Zentren) bzw. rein ambulant arbeitende Kooperationszentren (Typ-C-Zentren) als erforderlich für eine bestmögliche Versorgung von Betroffenen definiert [3].

Die Typ‑A Zentren, sogenannte Referenzzentren für SE, verstehen sich als Anlaufstelle für ärztliche Kollegen und Patienten. Sie sind behilflich bei der Suche nach geeigneten Ansprechpartnern innerhalb des Zentrums/Klinikums oder auch bei der Vermittlung von anderen Kompetenzzentren und Selbsthilfegruppen. Der Bedarf an diesen Angeboten ist in den vergangenen Jahren gerade bei SE erheblich gewachsen und wird gedeckt durch eine selbst für Betroffene und Fachleute überraschend positive, dynamische Entwicklung und Ausweitung der Diagnostik, Versorgung und spezifischen Behandlungen.

Die Abklärung einer unklaren Symptomatik und des Verdachts auf eine SE erfolgt durch die krankheitsübergreifend arbeitenden Referenzzentren bzw. Typ-A-Zentren. Sie ziehen je nach Bedarf Unterstützung durch lokale Experten im Rahmen von Konsilen oder Fallkonferenzen hinzu. Derzeit existieren in Deutschland an Universitätskliniken ca. 30 Zentren dieser Art. Aufgrund ihrer geringen Anzahl können die Wege zwischen den einzelnen Zentren weit sein. Betreuende Ärzte vor Ort und regionale Kliniken übernehmen daher einen (großen) Teil der Versorgung und können sich mit den Zentren in Verbindung setzen.

Hierfür ist ein enger Austausch, ggf. auf der Basis gemeinsamer Fallbesprechungen, erforderlich. Der Gemeinsame Bundesausschuss (G-BA) hat Regelungen zu den besonderen Aufgaben von Zentren für SE veröffentlicht, zu denen u. a. interdisziplinäre Fallkonferenzen und die Prüfung von Patientenakten für andere Einrichtungen gehören.

Für eine überregionale Zusammenarbeit bei SE wurden in den vergangenen Jahren mehrere IT-Systeme entwickelt und erprobt, die ein organisationsübergreifendes Patientenmanagement, Konsile und auch Fallkonferenzen erlauben. Basis dieser IT-Systeme stellt eine Elektronische Patientenakte (EPA) dar, in der alle Daten eines Patienten von den Beteiligten erfasst und den Behandlern über ein an die Anforderungen angepasstes Rechte- und Freigabemanagement bereitgestellt werden.

In diesem Beitrag möchten wir die Möglichkeiten und Grenzen von EPA am Beispiel der beiden Pilotprojekte „BASE-Netz“ und „TRANSLATE-NAMSE“ vorstellen, die sich mit der Implementierung von EPA für Menschen mit Seltenen Erkrankungen befasst haben. Die Erfahrungen bzgl. Machbarkeit und Akzeptanz aus dem Praxiseinsatz werden zusammengefasst und Schlussfolgerungen für die Entwicklung der EPA in Deutschland gezogen.

## Definitionen, Funktionen und Integration Elektronischer Patientenakten

Eine Patientenakte im Allgemeinen ist eine Sammlung von medizinischen Dokumenten bzw. Informationen zu einem Patienten. Eine EPA stellt die digitale Abbildung einer Patientenakte dar. Gemäß Fünftem Buch Sozialgesetzbuch (SGB V) § 341 ist sie eine „versichertengeführte, elektronische Akte, die den Versicherten von Krankenkassen auf Antrag zur Verfügung gestellt wird“. Mit ihr sollen „Informationen, insbesondere zu Befunden, Diagnosen, durchgeführten und geplanten Therapiemaßnahmen sowie zu Behandlungsberichten, für eine einrichtungs-, fach- und sektorenübergreifende Nutzung für Zwecke der Gesundheitsversorgung, insbesondere zur gezielten Unterstützung von Anamnese und Befunderhebung, barrierefrei elektronisch bereitgestellt werden“. Eine patientengeführte EPA verwaltet der Patient selbst und steuert eigenständig den Zugriff externer Gesundheitsorganisationen und seiner Angehörigen auf diese. Der Patient kann die Inhalte verändern und anpassen, z. B. Dokumente löschen, eigene Dokumente einstellen, Zugriffe sperren, ohne dass eine Organisation dies kontrollieren bzw. korrigieren kann. Man spricht auch von einer Gesundheitsakte, da die EPA nicht nur im Krankheitsfall genutzt werden kann (z. B. als Impfnachweis, Notfallausweis; [4]).

Neben der Definition der EPA als „patientengeführte Akte“ existieren zudem Definitionen, nach denen eine EPA von einer Organisation (z. B. Arztpraxis, Krankenhaus) geführt werden kann [4, 5]. Man spricht demnach von einer „organisationsgeführten EPA“, wenn eine Organisation die EPA zum Patienten bereitstellt und darin patientenbezogene Informationen sammelt. Sie kann intern in der Organisation genutzt oder organisationsübergreifend externen Organisationen, Patienten und Angehörigen bereitgestellt werden. In der Literatur wird diese Form der EPA als „institutionsgeführte Akte“ oder „Arztakte“ bezeichnet. Der Vollständigkeit halber ist auf das Konstrukt einer Elektronischen Fallakte (EFA) hinzuweisen, wenn die Daten „einem einzelnen Fall zugeordnet werden“ [4, S. 2]. Auf sie können die Behandler, die jeweils an einem Fall beteiligt sind, organisationsübergreifend, zweckbezogen und datenschutzkonform Zugriff erhalten.

Für eine organisationsgeführte EPA benötigt die Organisation die Zustimmung des Patienten. Zudem wird für jede Freigabe und anderweitige weiterführende Nutzung außerhalb des bisherigen Patienteneinverständnisses eine Zustimmung benötigt. Die Organisation erhält damit alle Befugnisse für die EPA und kann alle Inhalte so verwalten, wie es den Aufgaben am zuträglichsten ist.

Eine EPA verfügt grundsätzlich über Basisfunktionen zu Aktenverwaltung und Freigabemanagement. Zusatzfunktionen wie Formular‑, Aufgaben- und Workflowmanagement, Chat, Videokommunikation, Datenaustausch und viele weitere Funktionen lassen sich mit ihr verbinden. Fremdsysteme wie Krankenhausinformationssysteme (KIS), Praxisverwaltungssysteme (PVS) und Forschungsregister sind über eine Datenintegration anbindbar, um Daten aus der EPA bereitzustellen oder Daten in die EPA zu speichern. So lässt sich auf Basis einer EPA ein ganzheitliches IT-System, eine sogenannte E‑Health-Anwendung, zu Patientenmanagement, Diagnose, Behandlung etc. und zur Vernetzung von Organisationen zur gemeinsamen Patientenbetreuung einrichten. Ähnlich verhält es sich bei Gesundheits-Apps für Patienten, die eine patientengeführte EPA zur Patientendatenverwaltung im Hintergrund einsetzen.

Zu einem Patienten, der von mehreren Organisationen wie Krankenhäusern und Arztpraxen behandelt wurde, sind somit schon eine Vielzahl an organisationsgeführten EPA im Einsatz. Benutzt der Patient Gesundheits-Apps, führt er zudem auch mehrere patientengeführte EPA mit sich. Hinzu kommen die herkömmlichen Papierdokumente, wie z. B. Arztbriefe, Laborbefunde, Radiologiedaten, die der Patient von seinen Behandlern überreicht bekommt.

Es gibt somit nicht *die* eine Elektronische Patientenakte, sondern eine Vielzahl an EPA bei den unterschiedlichen Leistungserbringern, den Kostenträgern und beim Patienten selbst. Dies führt somit zur Problematik, dass in all diesen EPA wertvolle Informationen für Versorgung und Forschung liegen können, die von jeder Organisation neu erfasst werden müssen.

Um die Daten aus den unterschiedlichen EPA zu verknüpfen, gibt es i) die zentrale Integration mit Zusammenführung der Daten in eine zentrale Datenbank oder ii) die verteilte Integration mit dezentraler Verknüpfung der Daten, die in den Datenbanken der eingesetzten IT-Systeme liegen. Diese beiden Strategien werden bereits von zahlreichen Initiativen in Deutschland aufgegriffen:

- Auf Basis des Gesetzes zur Modernisierung der gesetzlichen Krankenversicherung (GKV-Modernisierungsgesetz) und des Gesetzes für sichere digitale Kommunikation und Anwendungen im Gesundheitswesen (E-Health-Gesetz) erfolgte in Deutschland die Einrichtung der Telematikinfrastruktur (TI) inkl. Medikationsplan, Notfalldatensatz, E‑Rezept, elektronische Arbeitsunfähigkeitsbescheinigung (eAU) und vieler anderer Dienste. Daran eng gekoppelt ist das Interoperabilitätsverzeichnis VESTA (Verzeichnis für informationstechnische Standards im Gesundheitswesen), in dem alle IT-Standards erfasst werden. Jede Einrichtung soll so eine EPA aus ihrem Primärsystem (z. B. KIS oder PVS) mit der TI verbinden und sich mit allen an der TI angeschlossenen Einrichtungen vernetzen können.
- Das Patientendaten-Schutz-Gesetz (PDSG) legte die Bereitstellung einer EPA für jeden Versicherten durch die Krankenkassen zum 01.01.2021 fest. Damit möchte man eine zentrale Zusammenführung aller Daten der Patienten in ihre EPA ermöglichen. Dabei liegt die Kontrolle der Daten beim Patienten.
- Mit der vom Bundesministerium für Bildung und Forschung (BMBF) geförderten Medizininformatik-Initiative wurde der Aufbau von Datenintegrationszentren begonnen. Ziel ist die Entwicklung von Standards, um Daten aus Versorgung und Forschung an den Universitätskliniken für eine vernetzte Forschung zusammenführen zu können. Diese Standards können darüber hinaus auch für EPA genutzt werden.
- Mehrere medizinische Gesellschaften und Initiativen setzen bereits epidemiologische und klinische Forschungsregister (z. B. Krebsregister, Traumaregister, Notaufnahmeregister) zur Integration der Daten aus verschiedenen Gesundheitsorganisationen ein, bei denen zentral Daten auf Basis eines definierten Datenschemas zusammengeführt und ausgewertet werden. Die Dokumentation und die Datenübermittlung in ein entsprechendes Register sind gesetzlich geregelt (z. B. Krebsregistrierung) bzw. an eine Zertifizierung (z. B. als Traumazentrum) gebunden. Die Organisationen exportieren gemäß einem Datenschema die Daten aus ihren IT-Systemen und übermitteln diese an das Register. Über digitale Fragebogensysteme und Patienten-Apps wird zudem erprobt, wie Patienten selbst ihre Daten zur Forschung übermitteln können.
- Zahlreiche Forschungs- und Versorgungsprojekte untersuchen die IT-basierte Vernetzung und Versorgung in unterschiedlichen Anwendungsbereichen und für verschiedene Indikationen, z. B. bei SE, Schlaganfall, Herzinsuffizienz, Parkinson, Brustkrebs u. v. m. Eine Vielzahl dieser Projekte wird durch den Innovationsfonds des G‑BA gefördert. Voraussetzung für die in den Projekten eingesetzten IT-Systeme ist die Anbindung an die TI.

Die grundlegende Anforderung an eine EPA ist das Vorhandensein einer standardisierten Datenstruktur, um eine möglichst hohe Interoperabilität der diversen EPA sicherzustellen. So lassen sich Patientendaten zwischen EPA ohne Mehraufwand für Migration bzw. Transformation austauschen. In Deutschland bietet sich hierfür der Standard „FHIR“ (Fast Healthcare Interoperability Resources) der internationalen Organisation „HL7“ an, den Hersteller mithilfe moderner Entwicklungswerkzeuge in ihren Softwaresystemen umsetzen können.

## BASE-Netz

Das Projekt „BASE-Netz“ (Netz des Bayerischen Arbeitskreises Seltene Erkrankungen; www.base-netz.de) hat das Ziel, die 6 bayerischen universitären Zentren für SE in Augsburg, Erlangen, München (Ludwig-Maximilians-Universität und Technische Universität), Regensburg und Würzburg mithilfe einer EPA und eines Workflowmanagementsystems zu vernetzen. Das Projekt wird vom Bayerischen Staatsministerium für Wissenschaft und Kunst von 2018 bis 2022 gefördert. Im Projekt unterstützt das Zentrum für Telemedizin Bad Kissingen (ZTM) als technischer Partner bei Anforderungsanalyse, Konzeption, Auswahl von Softwaresystemen und Inbetriebnahme. In enger Zusammenarbeit konfiguriert das ZTM die Workflows und Formulare für die Zentren und bringt sich im Betrieb als „Kümmerer“ mit Support und Projektmanagement ein.

### Technische Umsetzung einer einrichtungsgeführten EPA

Basis der Plattform ist eine EPA, auf die die Betroffenen selbst bzw. ihre gesetzlichen Vertreter, die Angehörigen, die überweisenden bzw. behandelnden Ärzte vor Ort und das betreuende Zentrum für SE Zugriff erhalten. Mithilfe eines Workflowmanagementsystems werden die Anmeldung der Patienten, die weitere Datenerfassung und die Kommunikation zwischen den Beteiligten koordiniert. Die Plattform wird ergänzend zu den Klinikinformationssystemen der Zentren genutzt, um auch standortübergreifend Daten austauschen zu können. Mittels Formulare können in die EPA alle relevanten Daten zur Krankheitsgeschichte eingegeben und alle medizinischen Dokumente wie Arztbriefe, Bilddateien (z. B. Röntgenbilder) oder Medikationspläne hochgeladen werden. Die EPA ist dem Zentrum zugeordnet, das die Anmeldung erhält. Sie kann von einem Zentrum an Externe zeitlich befristet freigegeben werden, um ihnen die für sie relevanten Daten zugänglich zu machen und die Möglichkeit zu geben, formularbasiert Daten einzugeben.

**Figure 1:**
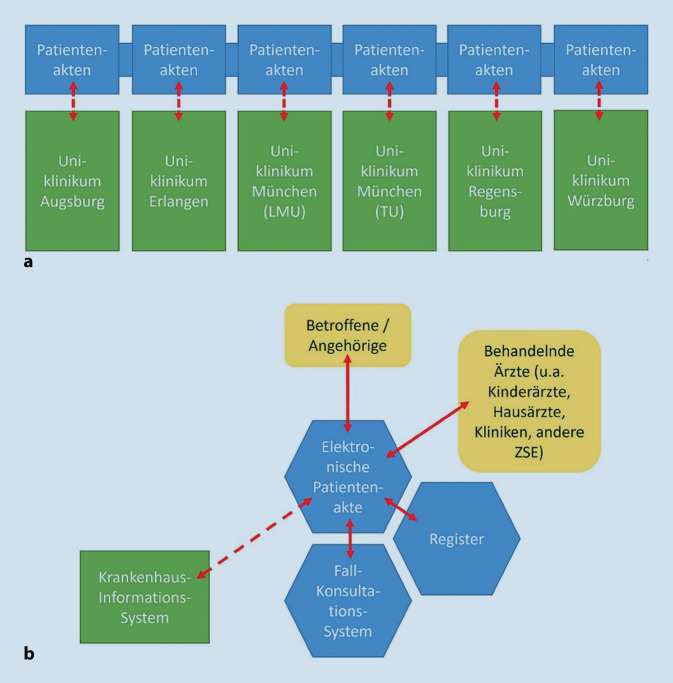

Einen Überblick über die Struktur und die digitalen Komponenten von BASE-Netz gibt Abb. 1 [6]. Die Abläufe der Dokumentenführung sind nach vordefinierten Schritten und „Meilensteinen“ organisiert. Beispielsweise kann bei der Abklärung einer Diagnose eine Rückmeldung zum aktuellen Stand an den Patienten bzw. seine Familie erfolgen und gleichzeitig ein einheitlich konsentierter Ablauf eingehalten werden. Es kann eine Bündelung aller Beteiligten aus Unikliniken und anderen Einrichtungen außerhalb der Zentren gelingen. Bei Einverständnis des Patienten bzw. seiner Sorgeberechtigten lässt sich die komplette EPA zur weiteren Betreuung an eine der anderen bayerischen Unikliniken übertragen.

Weiterhin werden aus dem BASE-Netz-Portal Daten für ein BASE-Netz-Register verfügbar. Dieses Register beinhaltet u. a. die Daten, die von der Europäischen Kommission oder im Rahmen eines französischen Konsensus als Kerndatensatz für SE definiert wurden [7, 8]. Damit soll BASE-Netz auch die kooperative Forschung der Netzwerkpartner unterstützen. Zum einen können über das Register passende Patienten für gemeinsame klinische Studien der Partner identifiziert werden. Zum anderen können die Unikliniken bei Studien zu gezielten klinischen Fragestellungen, wie z. B. nach dem Wert genetischer oder bildgebender Diagnostik bei SE, auf Einzelpatientenebene zusammenarbeiten und auf etablierte und datenschutzkonforme Abläufe zurückgreifen.

### Erfahrungen aus der Pilotierung

Auch wenn das Projekt noch mitten in der Umsetzung steht, können erste Erfahrungen festgehalten werden. Die Implementierung konnte erfolgreich durchgeführt und das Netzwerk zum 23.02.2021 in Betrieb genommen werden. Die webbasierte Anmeldung führt der Patient selbst durch. Insgesamt konnten bislang 701 Patienten eingeschlossen und betreut werden. Von diesen Patienten haben 525 die Online-Anmeldung genutzt. Insgesamt wurde 1271-mal eine Freigabe erteilt (451-mal für den Upload von Befunden, Arztbriefen, Bildern etc., 261-mal für Aufnahmeformulare, in die die Patienten Informationen zu ihrer Krankheitsgeschichte eingetragen haben, und 149-mal für eine Epikrise des behandelnden Arztes). Beim Upload wurden 276 DICOM-Dokumente und 2757 weitere Dokumente eingestellt.

Ergänzend zu der einheitlichen Vorgehensweise konnte jedes Zentrum eine individuelle Anpassung seiner Formulare (z. B. Logo, Einwilligungstexte) vornehmen. Technisch möglich wäre auch eine individuelle Anpassung der Workflows für jede Einrichtung, was aufgrund der gewünschten Einheitlichkeit zunächst keine Rolle spielte. Die einheitliche Struktur für die Datenerhebung wurde deshalb beibehalten. Dass weitere Zentren in die Struktur aufgenommen werden können, zeigt die nachträgliche Anbindung des Universitätsklinikums Augsburg innerhalb der Projektlaufzeit. Während das Projekt schon gestartet und die IT-Systeme in den bisherigen 5 Zentren zum Einsatz gebracht wurden, wurde in Augsburg erst ein Zentrum für SE eingerichtet. Nach Klärung der Rahmenbedingungen (u. a. Datenschutz, Auftragsverarbeitungsvertrag) erfolgten die Schulung und Einrichtung im Jahr 2022. Die Anbindung weiterer Typ-B-Zentren und Kooperationskliniken ist noch in Planung.

Ein Netzwerk wie BASE-Netz bedarf der kontinuierlichen Koordination und Abstimmung mit den Anwendern in den Zentren. Daher wurde ein monatliches „Key-User“-Treffen eingeführt. Dabei tauschen die Zentren standortübergreifend ihre Erfahrungen in der Routine aus und stimmen ihre Anpassungswünsche an Workflows und IT-Systeme ab. Einige Verbesserungsvorschläge wurden aufgegriffen, u. a. die Vereinfachung des Datenmanagements, die Anpassung von Formularen und Workflows an die tatsächlichen Anforderungen im Live-Betrieb, das Angebot regelmäßiger Nachschulungen bei Personalwechsel in den Zentren sowie die Rückmeldungen auf neue Updates.

Es zeigt sich im bisherigen Projektverlauf, dass die Anmeldung, die EPA und die Workflows von großer Bedeutung für die Versorgung sind, während sich die zusätzlich eingerichtete Videokommunikation zwischen den Zentren als sekundär erweist und nur selten für die Kommunikation mit Patienten verwendet wird.

Mit BASE-Netz konnte eine strukturierte EPA mit vordefinierten, vereinheitlichten Formularen bereitgestellt werden. Zudem wurde die Möglichkeit geschaffen, die EPA zwischen den Zentren auszutauschen und gezielt Leseberechtigungen für Konsile zu vergeben. Die formularbasierte Online-Anmeldung an einem der 6 beteiligten Zentren mit Eingabe von strukturieren Daten und Upload von Dokumenten wurde bereits innerhalb der ersten 12 Monate von Patienten, Angehörigen und Ärzten häufig (*n* > 2750) genutzt. Konsentierte Abläufe zwischen den Zentren legen „Meilensteine“ fest, die dem Anmelder Rückmeldung zum aktuellen Stand der Bearbeitung geben. Sie werden als hilfreich wahrgenommen und führen zu einer einheitlichen Datenstruktur, durch die für Forschungsfragen ein Register angebunden werden kann.

Der automatisierte Abgleich von Neuanmeldungen mit bestehenden Patientenakten vereinfacht die Erkennung von Mehrfachanmeldungen und deren Meldung an die betroffenen Zentren, um eine einrichtungsübergreifende Abstimmung zu ermöglichen. Aufgrund der einheitlichen Struktur und auf Basis eines Kooperationsvertrags ist die Anbindung weiterer Zentren dank einrichtungsübergreifender EPA, Workflows und Teamkoordination möglich. Dabei ist bzgl. der Lizenz- und Betriebskosten zu prüfen, wie eine möglichst preiswerte Infrastruktur etabliert werden kann. Je mehr Zentren die Plattform nutzen, umso besser lassen sich die Kosten untereinander aufteilen.

Daher stand in der Konzeption ein datenschutzkonformes Vorgehen im Vordergrund, um basierend auf einem sogenannten Mandantenkonzept mit wenig Aufwand die Hinzunahme weiterer Zentren mit eigener EPA und den identischen Workflows und Formularen einrichten zu können. Die Erweiterbarkeit auf andere Indikationen für die vernetzte Forschung und Versorgung ist theoretisch vorhanden, ist aber in Folgeprojekten mit anderen Fachbereichen zu prüfen und zu belegen. Es ist zudem noch auszuwerten, welche Funktionen tatsächlich in der Routineversorgung genutzt, welche Funktionen noch ergänzt und welche Daten für die vernetzte Forschung nutzbar gemacht werden können.

## TRANSLATE-NAMSE

Dieses Versorgungsprojekt „TRANSLATE-NAMSE“ wurde von April 2017 bis September 2020 durch den Innovationsfond des G‑BA für neue Versorgungsformen in der gesetzlichen Krankenversicherung gefördert [9–11]. Daran nahmen neben 9 universitären Zentren für SE 2 Krankenkassen (AOK Nordost und Barmer GEK) und die Allianz chronischer seltener Erkrankungen (ACHSE e. V.) teil. In dem Projekt werden die Maßnahmen des Nationalen Aktionsplans für Menschen mit SE (NAMSE) umgesetzt und ihre Wirksamkeit von den unabhängigen Institutionen Berlin School of Public Health und Zentrum für Evidenzbasierte Gesundheitsversorgung Universitätsklinikum Dresden (ZEGV) evaluiert.

In diesem Beitrag wird die Umsetzung des Projekts TRANSLATE-NAMSE im Zentrum für Kinder- und Jugendmedizin des Universitätsklinikums Heidelberg beschrieben. Hier wurden von Dezember 2017 bis Februar 2020 Patienten mit SE betreut. Ein schriftliches Einverständnis der Sorgeberechtigten und, falls möglich, der Patienten selbst wurde eingeholt. Das Projekt war von der lokalen Ethikkommission begutachtet worden (Aktenzeichen S‑499/2017).

Definierte SE wurden zunächst entsprechend der aktuellen Leitlinien der AWMF diagnostiziert, dazu gehörten angeborene Stoffwechselerkrankungen und Hypothyreose, adrenogenitales Syndrom (oder klinisch diagnostizierte Störungen der Geschlechtsentwicklung), primäre Immundefekte, Autoinflammationserkrankungen oder seltene Hämoglobinopathien. Anschließend wurden diese Patienten mittels der im Projekt entwickelten multiprofessionellen Versorgungspfade entsprechend den aktuellen Leitlinien betreut. Außerdem wurden Patienten ab einem Alter von 16 Jahren in die Erwachsenenmedizin weitergeleitet (sog. Transition; [12]).

### Technische Umsetzung einer patientengeführten EPA

Seit über 10 Jahren wird am Universitätsklinikum Heidelberg (UKHD) die persönliche, einrichtungsübergreifende Gesundheits- und Patientenakte (PEPA) entwickelt und angewendet [13]. Die PEPA wird vom UKHD gehostet und wurde in einen separaten Netzbereich ausgelagert, um einen sicheren Dokumentenaustausch zu ermöglichen. Sobald der Patient in die Nutzung der PEPA einwilligt, wird dies im KIS dokumentiert, die Patientendaten werden an die PEPA übermittelt und es wird eine Akte angelegt. Vorhandene Dokumente, wie Arztbriefe, definierte Laborbefunde und radiologische Befunde werden automatisch an die PEPA gesendet. Der betreuende Arzt sieht beim Aufruf des KIS, welche seiner Patienten in die PEPA eingewilligt haben, und kann bei Bedarf weitere Dokumente manuell an die PEPA senden. Die Datenhoheit liegt ausschließlich beim Patienten. Dieser entscheidet, welcher Arzt oder welche Institution Einsicht in seine PEPA haben darf. Darüber hinaus können niedergelassene Ärzte über ihr PVS an die PEPA angeschlossen werden. Patienten haben über eine eigens dafür entwickelte App Zugriff auf die PEPA [14]. Mittels eines PIN-Briefes wird der angelegte Account, welcher in der PEPA-App („phellow-App“) erstellt wurde, mit der PEPA-Akte verknüpft. Durch Aufruf des patientenbezogenen PEPA-Viewers können Dokumente der angeschlossenen Partner oder des Patienten eingesehen werden.

Im TRANSLATE-NAMSE-Projekt wurde den rekrutierten Patienten bzw. Sorgeberechtigten und den betreuenden Kinder- und Hausärzten der Zugang zu einer modifizierten Variante der PEPA angeboten, der sogenannten TRANSLATE-NAMSE PEPA. Dafür wurde zusätzlich eine separate Infrastruktur aufgebaut. Die Abtrennung des Netzwerks diente zur sicheren Anbindung der TRANSLATE-NAMSE-Partnereinrichtungen, welche über einen dedizierten Virtual-Private-Network-(VPN-)Tunnel auf die TRANSLATE-NAMSE PEPA zugreifen konnten. Zusätzlich zu dem Zugriff für die Klinikvertreter wurden webbasiert ein Patientenzugriff und ein Zuweiserportal zur Verfügung gestellt. Der Zugriff der Patienten bzw. Zuweiser erfolgte in einer eingeschränkten Portalansicht über einen Internetbrowser. Hier war lediglich ein lesender Zugriff auf die eigenen bzw. dem Patienten zugeordneten Dokumente gestattet. Die Patienten legten sich einen Account im Patientenportal an, welcher anschließend durch sie selbst mit der TRANSLATE-NAMSE PEPA verknüpft werden musste. Hierfür wurde den Patienten ein 31 Tage gültiger Verknüpfungscode zugesandt. Patientenzugriff und Zuweiserportal unterlagen einem separaten Sicherheits- und Datenschutzkonzept. Für das Projekt wurden beide an das Layout von TRANSLATE-NAMSE angepasst.

### Erfahrungen aus der Pilotierung

In TRANSLATE-NAMSE wurde das große Interesse von Patienten und Sorgeberechtigten an einer EPA deutlich. Knapp 95 % (422/445) der rekrutierten Patienten haben einer potenziellen Nutzung zugestimmt. Auch im Fragebogen (Rücklaufquote: 23 von 157 versendeten Fragebögen) gaben knapp 70 % (16/23) der Sorgeberechtigten an, dass ihnen das Vorhandensein einer elektronischen Akte wichtig bzw. sehr wichtig sei. Tatsächlich genutzt haben sie allerdings nur 27 % (117/422) der Patienten. Nach eigenen Angaben im Fragebogen haben 26 % (6/23) der Sorgeberechtigten diese häufig genutzt und 17 % (4/23) manchmal. Für die geringe Nutzung kann es verschiedene Gründe gegeben haben:

- Da die TRANSLATE-NAMSE PEPA ein Pilotprojekt war, wurden eilige Befunde weiter telefonisch an die Patienten bzw. Sorgeberechtigten übermittelt und Arztbriefe per Post versendet. Daher bestand keine zwingende Notwendigkeit der Nutzung.
- Die Sorgeberechtigten mussten sich aktiv einen Account im Patientenportal anlegen, welcher anschließend mit der TRANSLATE-NAMSE PEPA des Patienten durch die Sorgeberechtigten verknüpft werden musste. Dies stellt durchaus eine Hürde dar und setzt eine gewisse Technikaffinität und Digitalkompetenz der Sorgeberechtigten und Patienten voraus.
- Ein komfortabler webbasierter Zugang ist abhängig von der individuellen Übertragungsgeschwindigkeit des Internets sowie der Hardware.

Die zuweisenden niedergelassenen Ärzte nutzten die EPA mit 18,3 % (15/82) noch seltener als die Patienten bzw. deren Sorgeberechtigten. Ein möglicher Grund könnte gewesen sein, dass die Patienten bzw. Sorgeberechtigten direkt bei ihrem Ambulanzbesuch im Zentrum von den behandelnden Ärzten über die Vorteile der PEPA aufgeklärt wurden, während die Zuweiser aus Gründen des Datenschutzes von den Patienten zur Teilnahme motiviert wurden. Dadurch kam es wahrscheinlich zu Informationsverlusten und der unmittelbare Nutzen wurde nicht ausreichend ersichtlich. Zudem musste für dieses Pilotprojekt ein relativ hoher administrativer Aufwand betrieben werden, bis eine Teilnahme gewährt wurde, u. a. musste ein schriftlicher Vertrag zwischen Zentrum, Patienten und zuweisendem Arzt geschlossen werden. Weitere eher technische Gründe für die geringe Nutzung der EPA von den zuweisenden Ärzten:

- Im Rahmen dieses Pilotprojektes war keine tiefe Integration des Aufrufs der TRANSLATE-NAMSE PEPA in das PVS gegeben. Der Aufruf des webbasierten Zuweiserportals konnte deshalb nicht zuverlässig in den Praxisalltag integriert werden. Es entstand ein zusätzlicher Aufwand ohne spürbaren Mehrwert. Eine Vereinfachung der Anmeldung z. B. durch eine integrierte Single-Sign-on-Lösung im PVS könnte die Nutzer zukünftig stärker zum Absprung in die PEPA motivieren.
- Die zuweisenden Ärzte betreuten insgesamt nur sehr wenige – meist nur 1 oder 2 – Patienten, die eine TRANSLATE-NAMSE PEPA besitzen. Daher war hier der Aufwand in Relation zum tatsächlichen Nutzen hoch.
- Viele Praxen scheinen auf die Nutzung von EPA noch nicht vorbereitet zu sein. Eine im Rahmen des Projekts durchgeführte Befragung hat gezeigt, dass sich von 1500 befragten Haus- und Kinderärzten nur 122 von 204 antwortenden Ärzten (60 %) eine elektronische Vernetzung mit einem Zentrum für SE wünschen. Von diesen waren lediglich 42 % an einer einrichtungsübergreifenden elektronischen Gesundheits- und Patientenakte interessiert [15].
- Im Verlauf des Projekts konnte die technische Anbindung weiterer Projektpartner an die TRANSLATE-NAMSE PEPA noch nicht realisiert werden. Gezeigt werden konnte, dass eine Anbindung weiterer Kliniken im Testsystem möglich gewesen wäre.

Insgesamt war in diesem Projekt das Interesse an der Nutzung einer EPA bei den Patienten bzw. Sorgeberechtigten höher als bei den Mitbehandlern. Um die Akzeptanz zu erhöhen, müssen bessere technische Voraussetzungen geschaffen werden, wie schnellere Internetverbindungen und Integration in PVS.

## Schlussfolgerungen für den Ausbau der EPA in Deutschland

Dass der Einsatz einer EPA Vorteile bietet und datenschutzkonform einrichtungsübergreifend umsetzbar ist, haben die hier vorgestellten Projekte BASE-Netz und TRANSLATE-NAMSE demonstriert. Nach Campanella et al. [16] können EPA einen schnellen Informationsaustausch zwischen Behandlern, Patienten und Mitbehandlern ermöglichen und damit mittel- und langfristig eine höhere Therapieadhärenz, kürzere Hospitalisierung und insgesamt eine verbesserte Lebensqualität für die Patienten erzielen. Durch die zeitnahe Informationsbereitstellung kann sich deren Zufriedenheit erhöhen. Aufseiten der betreuenden Ärzte kann sich zusätzlich ein Rückgang der telefonischen Nachfragen ergeben.

Durch die in der Einleitung beschriebenen neuen gesetzlichen Rahmenbedingungen zur TI und EPA der Versicherten nach SGB V § 293a bzw. § 341 stehen zukünftig weitere technische Möglichkeiten der Vernetzung und Einbindung der Patienten zur Verfügung. Aus den Erfahrungen von TRANSLATE-NAMSE ließe sich so erhoffen, dass die Akzeptanz bei den niedergelassenen Praxen steigt, da keine zusätzliche Anbindung notwendig wird und der Patient über die patientengeführte EPA die („Daten“‑)Kommunikation und Freigabe aus eigenem Interesse übernimmt. Das unterstreicht auch die sehr positive Resonanz der Patienten auf das Projekt und das digitale Angebot. Unerlässlich sind stetige Aufklärungen und Transparenz, um insbesondere über datenschutzrechtliche Fragen zu informieren. Auch sollten Schulungen und Unterstützung angeboten werden, um die digitale Kompetenz zu fördern.

In der Theorie könnte der Eindruck entstehen, dass durch die Einrichtung der TI alle Akteure einfach ihre Systeme anbinden und beliebig miteinander vernetzen können. Für die Praxis ist allerdings erst zu prüfen, wie die EPA der Einrichtungen und die EPA der Patienten verknüpft und alle relevanten Daten für Versorgung und Forschung sowohl dem Patienten selbst als auch den beteiligten Einrichtungen zur Verfügung gestellt werden können. In BASE-Netz ergibt sich durch den Workflow-basierten Ansatz die Anforderung, dass die Prozesse auch einrichtungsübergreifend IT-basiert unterstützt werden müssen, um eine Übersicht über die Patientenversorgung („Meilensteine“) zwischen den Beteiligten zu erhalten. Eine reine Datenübermittlung, bei der jede Einrichtung eigene Softwaresysteme wie KIS und PVS einsetzt, wird einen übergreifenden Workflow nicht abbilden können. Es ist daher zu prüfen, wie übergreifende Workflowmanagementsysteme und einrichtungsübergreifende EPA mit der TI verknüpft werden können. Ein Ansatz hierfür kann neben der Entwicklung von Interoperabilitätsstandards die Entwicklung von Standards für Workflows sein, wie man sie bei dem Standardisierungsgremium „Integrated Health Enterprise“ (IHE) bereits für Radiologie und Kardiologie entwickelt hat. Wichtig hierfür ist auch die Einrichtung eines eindeutigen Patienten-Identifikators, was noch nicht vollständig durch die TI gegeben ist. In BASE-Netz wurde daher ein eigener Master-Patient-Index implementiert, um Mehrfachuntersuchungen und Verwechslungen zu vermeiden.

## Fazit

Beide Projekte haben die technische und rechtliche Machbarkeit für den praxisnahen Einsatz einer EPA für die Versorgung und Forschung zu Seltenen Erkrankungen aufgezeigt. Aufseiten der Patienten sind ein hohes Interesse und Bereitschaft für die Nutzung einer EPA zu beobachten. Die geplanten Arbeitsprozesse an den Zentren konnten erfolgreich umgesetzt und eine einheitliche Datenstruktur etabliert werden. Für externe Mitbehandler ist der Aufwand für die Teilnahme zu vereinfachen und der Mehrwert noch weiter auszuarbeiten. Im Projekt BASE-Netz ist es geplant, die entwickelte Plattform weiter mit Komfortfunktionen auszubauen und sichere, datenschutzkonforme Cloud-Plattformen auch anderen Zentren und Kooperationseinrichtungen anzubieten. Der Ansatz der einrichtungsübergreifenden vernetzten EPA lässt sich, einmal etabliert, auch für weitere Indikationen in der Versorgung von Menschen mit chronischen Erkrankungen ausbauen.
